# Supplementary material for: Calmodulin controls spatial and temporal specificity of calcium-induced calcium release
Source: PLoS Comput Biol. 2026 Sep 10;22(9):e1013752. doi: 10.1371/journal.pcbi.1013752 (PMC13581220; doi:10.1371/journal.pcbi.1013752)
Supplement: Supplementary file 1 [file pcbi.1013752.s005.pdf]

Revision letter

“Calmodulin controls spatial and temporal specificity of calcium-induced calcium release”

Dear Editors and Reviewers,

We would like to thank you for your detailed reviews of our manuscript, which greatly helped to improve it. We have rewritten the manuscript according to your comments. Please find our response below.

Thank you for your consideration of this manuscript.

Sincerely,

Joanna Jędrzejewska-Szmek and Kim T Blackwell

Reviewer #1: The investigation of CICR in control and experimental conditions -aging, AD- is relevant and original. The model explores different scenarios and investigates the role ER Ca2+ release, RyR2, PMCA and CaM in this process. However, the authors need to address some issues.

Justify why a stochastic model is really needed. Compare with a deterministic solution.

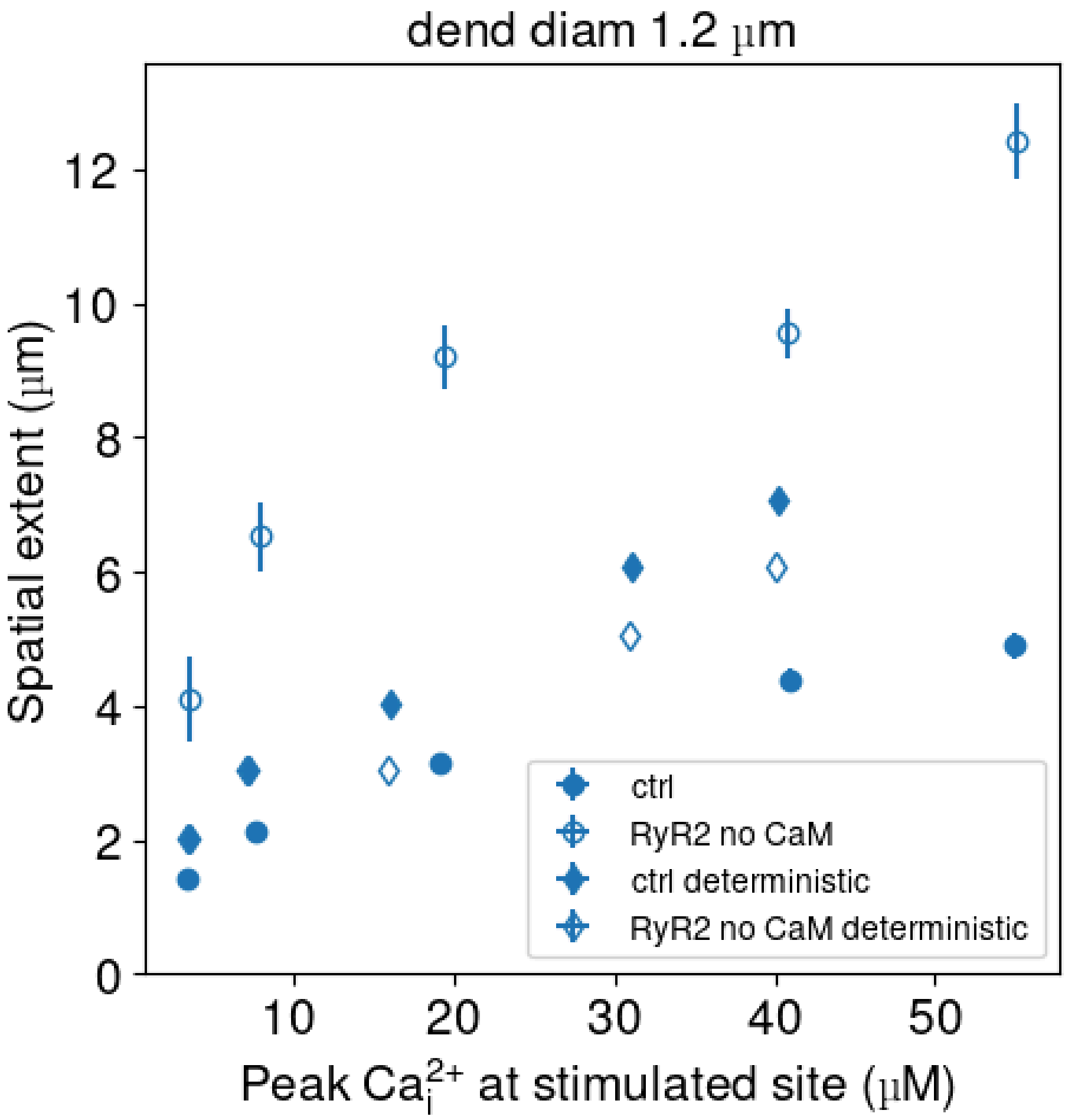

We used stochastic methods, because we had low copy numbers. As explained in the discussion, lines 431-443, a stochastic simulation method is better suited for dynamical systems with low copy numbers [Jędrzejewska-Szmek et al., 2023] and in such systems deterministic models might yield different predictions than stochastic models [Friedhoff et al., 2021]. One of the differences is that deterministic rate laws depend on concentrations whereas stochastic rate laws depend on the number of possible combinations of reacting species. With high copy numbers stochastic and deterministic models will yield similar results, whereas with low copy numbers the reactions might differ substantially.

We did run a subset of the simulations using deterministic solvers (using the 1.2 um dendrite), as shown in the figure above. However, the results show a difference, especially for the calmodulin inhibited RyR2 condition. Note that the system is very stiff, which makes accurate deterministic simulations problematic.

The work mention the role of Ca2+ in learning and memory, but the model does not reproduce learning an memory phenomena. How to truly link the model results with

learning and memory?

We added the following paragraph to the Discussion, which also addresses Reviewer 3's comments on RyRs in spines (lines 404-418): "The influence of calmodulin and RyR2 on calcium dynamics is critical because learning, memory and many other processes in the brain are controlled by calcium dynamics. Dendritic calcium plays a crucial role in behavioral time scale plasticity, a very powerful form of synaptic plasticity requiring a prolonged dendritic calcium elevation, which underlies tuning of spatial features in dendrites and is capable of producing place cells [Bittner et al., 2017]. Interestingly, blocking mitochondrial calcium buffering, and hence increasing calcium release from the ER, led to higher stability of place cells [O'Hare et al., 2022]. Dendritic calcium is strongly correlated with spine calcium [Majewska et al., 2000] with RyR3s (activated by calmodulin) on the spine endoplasmic reticulum (spine apparatus) transducing spine calcium signals to the dendrite [Breit et al., 2018, Basnayake et al., 2019]. Calmodulin acting on RyRs differentially regulates the calcium transients in the dendritic branch: leading to an amplification of calcium transients from individual spines and restricting their spread in the dendrite leading to larger spatial and temporal specificity of calcium signaling in the dendrite. Thus, RyRs can influence learning and memory through their contribution to dendritic and spine calcium transients."

Check throughout for typos and English errors. To mention some: 'disinhibted', pg. 9. 'AD we tested …', pg 12.

We have carefully reviewed the manuscript for any English errors and run a spelling check.

Check labels and titles in all figure: Peak Ca or peak Ca^2+? Ca or Ca^2+?
Make sure fonts are consistent in the figures.

Fig. 15: Paradigm is upside-down.
Fig 2: Use the proper superscript for 2+ in Ca2+

We have applied all these changes to the figures.

There is an overlap in the header of the tables 2, 7 and 8 between kf and kr.

We have fixed the overlap in the header in tables 2, 7 and 8 (Tables 1, 6, 8 in the revised manuscript).

Justify the inconsistent units between tables for kf. Table 4: kf(nM/ms) Table 2 kf(nM^-1 ms^-1). Table 6 kf(nM^4/ms). How are the different units handled in the program code?

The units were an error in latex rendering that has been fixed in the revised manuscript. In Table 4 and 5 kf is in nM^-1ms^-1 and in Table 6 in nM^-4 ms^-1. The power of nM units is determined by the number of reactants.

Reviewer #2: Major Comments:

1) The text of the paper, including but not limited to the Discussion, needs to be revised to eliminate any implication that the authors are simulating any changes or processes specific to Alzheimer's disease. Because, in fact they are not, all the listed changes in model parameters – altered RyR2-Cam binding, decreased PMCA pump activity, downregulation of SOCE and of T-type Ca channels – are generic to old age, not specific to AD. Indeed, some of the tables of model parameter values have columns listing old age values, but not columns listing different AD values. If the authors really want to claim they are simulating an AD condition, they need to extend the model and present additional simulations, by adding the effects of elevated beta-amyloid that they note in the Discussion.

The old age model and the AD model do represent two different conditions and we have edited the manuscript to clarify this. The old age model differs from the AD model in (a) the lack of SOCE and (b) higher buffering molecules, including Neurogranin. These differences are listed in Tables 2 and 7, and we also state those differences in the Results in lines 321-323 and in the Discussion in lines 511-513. In addition, the old age model results differ from the AD model results. Specifically, we have not observed calcium oscillations or elevated calcium in the old age model most probably due to the increased calcium buffering that is observed in old age (and not in Alzheimer's disease).

2) The authors say their model is multi-compartment, but do not give or sufficiently justify a description of these compartments. How is their submembrane compartment different from the rest of the dendrite, why does it have the given width? Are there simple exchange terms from this compartment to the rest? Figure 3 has a very odd depiction of the dendrite as a gold cylinder hovering between two planar plasma membranes. I think the dendrite is instead supposed to be a box as in Fig. 2, so fix Fig. 3. Also, from the author's preceding paper ref. 32, I guess the dendrite is divided into voxels. What is their size and shape? If NeuroRD does it automatically, still give an approximate description. Is diffusion modeled using the diffusion partial differential equation everywhere, or is it modeled by simple exchange terms at the boundaries of voxels with voxel interiors assumed homogenous, or is there both diffusion and exchange terms?

Yes, the dendrite is a box as in Fig. 2. We have removed Fig. 3 from the manuscript as it was giving inaccurate information.

We implemented the signaling pathways model (Fig 1) in 51 μm long dendrites with diameters of 1.2 μm, 2.4 μm and 6.0 μm representing thin, medium and thick dendrites, respectively (Fig 2). Each morphology was divided into voxels. Submembrane voxels, which allow for placing of membrane-associated molecules, were cubes of 0.5 μm × 0.4 μm × 0.6 μm (the same for all dendritic diameters). Cytosolic voxels were the same as submembrane voxels for the 1.2 μm model, cubes of 0.5 μm × 0.46 μm × 0.6 μm for the 2.4 μm model and cubes of 0.5 μm × 0.4615 μm × 0.6 μm for the 6.0 μm model. The submembrane voxels allow for placement of membrane associated molecules. The diffusion is simulated stochastically everywhere using diffusive coupling strength between all voxels. This is explained in (Jędrzejewski-Szmek and Blackwell, 2016), and is similar to that implemented

in (Lampoudi et al., 2009, Fange et al., 2010). We have improved the explanation of this morphology in the methods, lines 96-103.

Fange D, Berg OG, Sjöberg P, Elf J (2010) Stochastic reaction-diffusion kinetics in the microscopic limit. Proceedings of the National Academy of Sciences of the United States of America U.S.A. 107 (46): 19820-19825,

Jędrzejewski-Szmek Z, Blackwell KT (2016) Asynchronous τ-leaping. Journal of Chemical Physics, 144, 125104

Lampoudi S, Gillespie DT, Petzold LR (2009) The multinomial simulation algorithm for discrete stochastic simulation of reaction-diffusion systems. Journal of Chemical Physics 130(9):094104. doi: 10.1063/1.3074302.

3) The paper implies, at first reading, that the ER is modeled as distinct internal compartments with their own surfaces and internal volume. Figure 3, right panel, illustrates the model as if this was the case. But for this model variant (I comment below on the left panel) It looks like the authors distributed the variables RyR2, RyR2Ca, and CaER uniformly throughout (according to lines 74-76) a quarter of the dendrite, with this quarter consisting of one or more compartments with RyR2 and RyR2Ca volume-distributed. The authors did not, it appears, construct separate ER compartments with their own surface putting RyR2 on the surface of these compartments. And, given that the authors do not mention separate CaER variables for separate compartments, I think they only assume one internal ER compartment, which is contrary to what the figure shows. It is essential that the article text, and the figure in particular the location of RyR2, SERCA.., be clarified, and extended as necessary, so that no misleading material is present in this regard. It would also be good to give some detail about how existing data-based ER reconstructions compare with this model.

The modeling of the ER within the cytosol is indeed an extremely difficult problem. ER reconstructions show a continuous network of anastomosing membrane cisternae that form a parallel network with periodic larger compartments and plasma membrane-endoplasmic reticulum junctions (Benedetti et al., 2024). It is very difficult to model this fractal like geometry and an accurate model would require a particle-based approach which in turn would make it computationally impractical to model calcium dynamics in a 51 micron long dendrite. We were interested in the mesoscopic calcium dynamics and the dendritic spread of calcium transients along the dendrite viewing the dendrite more as a 1-dimensional structure. Hence, we decided to treat the ER as a uniformly distributed structure in the dendrite taking up 25 % of its volume, containing a separate population of calcium molecules diffusing within the ER more slowly (due to the ER tortuosity) than the cytosolic calcium molecules. The randomly placed ryanodine receptors allow for a release of ER calcium molecules to the cytosol, with flow of calcium down the concentration gradient proportional to the number of open RyR receptors. This is now explained in the methods section in lines , and in the discussion, lines 466-476.

We do not discuss the second variant of the ER morphology, because another reviewer suggested removing it altogether and we agreed that the other ER variant was confusing.

Benedetti L, Fan R, Weigel A, Moore AS, Houlihan PR, Kittisopikul M, Park G, Petruncio A, Hubbard PM, Pang S, Xu CS, Hess HF, Saalfeld S, Rangaraju V, Clapham DE, De Camilli P, Ryan TA, Lippincott-Schwartz J.(2024) Periodic ER-plasma membrane junctions support long-range Ca2+ signal integration in dendrites. Cell 188: 484-500.e22

4) The ER model in the left panel of Fig. 3, in which the RyR2s are all located "at the dendritic membrane", by which is meant either the lower or upper face of the model's submembrane compartment, seems unjustified. It looks like here the authors put a planar box, right below the submembrane compartment, taking up a quarter of the dendrite volume, with the RyRs and SERCA all on that upper face. In reality, even if an ER cisternae lies against the membrane, I don't think the RyRs and SERCAs would all be on that face, they should be on other faces of the ER compartment as well. Is there relevant data, has anyone showed by immunostaining or another method how RyR2 or SERCA is distributed within the dendrite? Anyway, the authors state later that simulations with this model variant are not significantly different from simulations with the model variant in the right panel. Thus, I think that model variant should be removed.

We have removed that model variant as stated above, and the RyRs are distributed uniformly.

5) The authors need to state if, and for how long, their model with basal parameters and starting from initial concentrations was equilibrated, prior to giving a stimulus.

We have added that information to the methods, lines 145-149. "All model variants were simulated without any stimulation to reach steady state (usually for 1000 s). Initial conditions of all the model species were recalculated based on the last 25 s of those simulations and the models were rerun without any stimulation to verify the stability of the steady state. If the steady state was not reached the procedure was repeated. For simulations of calcium wave induction only models in steady state were used."

6) References, or other justification for estimates, need to be provided for the parameter values in Tables 1, 3, and 5. For non-ER parameters some of this may already be in the authors' preceding paper, Ref. 32. If so make it clear to the reader, describing for which parameters references or justification is found in Ref. 32. But for parameters new to this model it needs to be done here.

All necessary references have been added in Tables 1, 3 and 5 (Tables 4, 2 and 7 in the revised manuscript). We also clarify when we used the data in the reference to tune the parameters.

7) It is stated that the simulations are performed stochastically, with use of the Gillespie algorithm. Is this true even for the most abundant molecules and fastest events, like calcium binding and unbinding from buffers? What about calcium diffusion. It appears the authors likely use a hybrid simulation scheme where (some or all) reactions are Gillespie and diffusion (at least) is not?

We have clarified the mention of Gillespie's algorithm in the discussion. In fact we used a spatial extension of Gillespie's tau leap algorithm, which dynamically selects between simulating an exact event or a leap event both for reactions and diffusion between voxels (Jędrzejewski-Szmek and Blackwell, 2016). This algorithm simultaneously improves both the efficiency and accuracy over prior tau-leap algorithms, and avoids statically partitioning the system. This algorithm is better suited for stiff systems, and systems with a mixture of low and high copy numbers. We clarify this in lines 177-179 (methods) and in the discussion, lines 439-443.

8) Tables 6 and 7 repeatedly use the phrase "adjusted to effect". Explain what this means. For example it could mean data shown in a particular figure of the referenced publication.

We added clarification of the effect in all relevant places. As you surmise, parameters were adjusted to fit particular figures in references; we now provide both the reference and the figure in the tables (Tables 5 and 6 in the revised manuscript).

9) I see one place, Figure 7, where the authors give a calcium stimulus amplitude, but it is in molecules injected, which is hard to relate to physiological stimulus amplitudes as commonly reported. Please compare the amplitudes to physiological stimulus amplitudes in some manner. What is the duration? On line 110 the authors say the authors increased calcium stimulus to take into account L-type channel upregulation. How much? But in fact, in the Discussion it says L type VGCCs were "not included in the model"? What about the T-type channel downregulation the authors say also occurs in aging?

We added two tables in the Methods section (Tables 9 and 10) providing stimulus details (amplitude, duration, stimulation site) linking the stimulus amplitude measured in injected molecules to calcium concentration observed in stimulated dendrite.

We did not include explicit voltage-gated channels, and instead included an estimate of calcium influx through those channels. Changes in the L-type channel expression were simulated as an increase in total injected calcium (Table 9. shows a comparison of synaptic-like activation stimulation in control model and old age models), emulating the change in synaptic activation of L type calcium channels. Calcium influx via T-type VGCC was included as a leak from the extracellular space (Leak molecule), to emulate a small amount of calcium influx. In the old age models we decreased Leak by 17 %.

The sentence in the discussion was rewritten to indicate that membrane potential activity (which activated L-types) was critical for the observed calcium wave.

10) During each simulation in the old age condition, when RyRs are only 50% saturated with CaM, this level of saturation is not a dynamic variable. In actuality, though, this level should vary because the binding of calcium to CaM is dynamic, calcium is varying, and apo-CaM and Ca-bound CaM should have different RyR2 affinities. If the authors don't want to add simulations, they need to acknowledge in

the Discussion that this is a model limitation, or else justify why it can be neglected. I guess if half of the RyR2s are actually normal and half can't bind CaM at all, then it could be neglected, but this should be better justified.

We agree that calmodulin binding to RyR2 should be dynamic. However, the binding of calmodulin to RyR is in fact very slow with the dissociation rate having t1/2 of 9 min in presence of calcium (Balshaw et al, 2001). We did a limited number of simulations, with dynamic binding of calmodulin to RyR during a transient calcium elevation and observed that new calmodulin did not bind to the RyR subunits due to the slow reaction rates. We have now added this justification to the Discussion, lines 452-460.

Balshaw DM, Xu L, Yamaguchi N, Pasek DA, Meissner G. (2001) Calmodulin binding and inhibition of cardiac muscle calcium release channel (Ryanodine Receptor)*. Journal of Biological Chemistry 276(23): 20144-20153

11) For Figure 1, the legend is not adequate. I'm not sure this figure shows all the pathways, given that this model builds on that presented in Ref. 32. In the previous Ref. 32 the authors have another similar figure of signaling pathways. Tell the reader; if they combine that figure with this Fig. 1, do they see all the pathways?

Figure 1 of the manuscript shows all the pathways of the model. We did not implement all the pathways of ref. 32, because that was beyond the scope of the project. Instead, we used only the pathways directly involved in controlling calcium dynamics, such as calcium binding proteins and cytosolic pumps. For example, we did not include the cAMP/PKA pathway because most of the experiments measuring calcium release do not add ligands for Gs coupled receptors. We did not add CamKII because its role in modulating RyR2 is unclear. In addition, we used simulations in the model of ref. 32 to determine how much calmodulin would be available were CamKII present. We have added a figure caption that explains all the pathways in Fig 1: "*Molecular pathways implemented in the model. Calcium flows into the cell through the calcium permeable channels in the cell membrane (denoted VGCC in the diagram and Leak in Tab. 1). Calcium is extruded into the extracellular space by the plasma membrane Ca2+ -ATPase (PMCA) and sodium calcium exchanger (NCX) and into the endoplasmic reticulum by the Sarco/endoplasmic Reticulum Calcium ATPase (SERCA). In the cytosol calcium is buffered by calbindin, indiffusible buffer (Buffer) and calmodulin (CaM). Apo-calmodulin (unbound to calcium) is buffered by neurogranin (Ng). Calcium flows from the ER to the cytosol through open ryanodine type 2 receptors (RyR2), which are activated by calcium and inhibited by calmodulin. In the ER calcium is buffered by calreticulin (CRT) and binds stromal interaction molecules (STIM), which serve as luminal calcium sensors. When calcium in the endoplasmic reticulum is too low, STIM molecules form dimers and bind calcium release-activated calcium channel protein 1 (Orai1) opening them and allowing calcium from the extracellular space to flow into the cytosol, where it would be transferred by SERCA to the endoplasmic reticulum, thereby replenishing it. The ORA1-STIM channels constitute store-operated calcium entry (SOCE).*"

12) Lines 275-277, there is a sentence, "This result is consistent with experiments showing that in hippocampal CA1 neurons IP3-sensitive calcium store refilling required ORAI1 channels, whereas ryanodine-sensitive calcium store refilling involved VGCCs". This sentence is not justified, because none of the simulations in

this paper were designed to specifically examine depletion of calcium stores or mechanisms for their refilling. Either remove this sentence or add additional, appropriate simulations carried out for a longer time during which ORA1I and VGCCs are separately inhibited.

This sentence has been removed.

13) Lines 79-82 as written imply two different models were used to describe the RyR2. This needs to be better explained. How is the model for the oxidized state (the Keizer Smith model) different from the model for the non-oxidized state? Can this difference be justified?

In old age calmodulin becomes oxidized. Baldshaw et al., (2001) showed that, in the oxidized conditions with cytosolic calcium levels, calmodulin affinity for RyR2 dropped twenty-fold, resulting in 50% RyR2s bound to calmodulin and hence inhibited. Based on this we divided the RyR2 population in two: one bound to calmodulin and consequently inhibited, and the other not bound to calmodulin. The two models differ considerably as can be seen in Fig. 2 of Xu and Meissner, 2004. The opening probability curve for the inhibited RyR2 (+1 uM CaM condition in Fig 2 of Xu and Meissner, 2004) is shifted towards higher calcium concentrations and the mean receptor open times are shorter with longer mean receptor closed time than the RyR2 unbound to calmodulin.

We have clarified our terminology regarding the old age model (which refers to the entire model and includes RyR2 in the presence of oxidized calmodulin) and the oxidized model (terminology no longer used), both in the methods (lines 92-94, 111-131) and in the results (lines 260-302).

Xu L, Meissner G. (2004) Mechanisms of calmodulin inhibition of cardiac sarcoplasmic reticulum Ca2+ release channel (Ryanodine Receptor). Biophysical Journal 86(2): 797-804

Minor Comments:

1) In Fig. 11b, it looks like the calcium waves always start at one end of the dendrite. Why don't some initiate in the middle, like the calcium sparks do?

We have changed Fig 11a (Fig 7a in the revised manuscript) and Fig 11b (Fig 7b in the revised manuscript) to one that shows more clearly that calcium waves have a slight curvature and do in fact begin in the middle of the dendrite.

We have added an inset expanding the time from 800-900 sec of 1.2 um dendrite (Fig 7d) to show that calcium waves can start in the middle of the dendrite and propagate in only one direction.

2) Tables should be numbered in the order in which they are introduced in the manuscript, and should be briefly described when first introduced (this is not done for Table 1).

We have reintroduced the tables accordingly in the method section.

3) The legend of Fig. 2 is not adequate. Give dimensions, including the submembrane compartment. The legend of Fig. 3 needs to be revised because the model is not actually implementing “parallel cisternae” as compartments with distinct surfaces.

We removed Fig. 3 from the revised manuscript.

We have rewritten the caption of Fig. 2. It now states:

“Dendritic morphologies used in simulations. We used three dendritic morphologies: thin dendrite (diameter of 1.2 μm), medium dendrite (diameter of 2.4 μm) and thick dendrite (diameter of 6.0 μm). All dendrites were 51 μm) long. Each morphology was divided into voxels, allowing two-dimensional diffusion. Submembrane voxels were cubes of 0.5 μm × 0.4 μm × 0.6 μm (the same for all dendritic diameters). Cytosolic voxels were the same as submembrane voxels for the 1.2 μm model, cubes of 0.5 μm × 0.46 μm × 0.6 μm for the 2.4 μm model and cubes of 0.5 μm × 0.4615 μm × 0.6 μm for the 6.0 μm model. For simulating synaptic activation calcium molecules were injected into submembrane voxels in the middle of the dendritic morphology (denoted EPSP stim on the diagram) or into the cytosol to an end dendritic voxel (denoted AP stim on the diagram).”

4) There are a number of minor grammar errors throughout the manuscript, go over it with either a human or AI editor.

We have re-edited the entire manuscript to eliminate grammatical errors.

5) Line 145, Fig. 4 should be Fig. 3.
6) The acronyms EPJ and SOCE need to be defined at first use.

SOCE is now defined in the Methods section at first use. EPJ is no longer used in the manuscript as we eliminated that model variant as suggested in Major Comments.

7) Is “spatial extent” calculated for Fig. 5 the same as for Fig. 6?

Yes, it is. We have added that information to the methods and to Fig. 6 (Fig 4 in the revised manuscript).

8) The formatting of the References has numerous inconsistencies.

This has been fixed.

9) Lines 138-140, you say 4 um is a theoretical limit for calcium transient spread. I don’t see how this can be a theoretical limit, it must be parameter value dependent.

This has been clarified. It is the theoretical limit calculated for high frequency stimulation (80 Hz). Other minor comments have also been addressed in the manuscript.

Reviewer #3: This manuscript describes the use of a computational model to explore the contribution of calcium-induced calcium release (CICR) from endoplasmic reticulum (ER) to calcium transients and resting state in neuronal dendrites. Calcium dynamics are crucial to neuronal function and synaptic plasticity, so understanding the many mechanisms that determine such dynamics is highly important. This current work provides a useful increment to our knowledge, detailing the amplitude, spatial extent and time course of dendritic calcium transients due to pseudo-synaptic stimulation, and the contribution of CICR to this. The effects of changes in CICR during aging and Alzheimer's disease are explored. The presentation of the work, however, could do with some improvement, as detailed below.

Introduction

Point the reader to Fig. 1 when ER first introduced eg around line 14.

We are concerned that referring the reader to Figure 1 in the second paragraph of the introduction would be confusing, because there are many molecules in Figure 1 that are not mentioned until the methods, and some of the molecules in that paragraph are not in Figure 1. We now refer the reader to reference [6] for more information and a figure portraying these molecules.

Methods

Line 111: Details of the calcium stimuli used are needed (amplitudes, time course). Are these stimuli reasonable approximations to what is expected from synaptic activation on a spine head? Does figure 2 indicate that the stimuli are from a point source at the location indicated?

A subsection of the methods section titled "Stimulation protocols" has been added (lines 144-176) with Tables 9 and 10 detailing specifics of the stimulation protocols. The relevant information also has been added to the end of the Fig. 2 caption: "For simulating synaptic activation, calcium molecules were injected into submembrane voxels in the middle of the morphology (denoted synaptic stim on the diagram). For simulating b-AP like stimulus, calcium molecules were injected into the cytosol to an end dendritic voxel (point stimulus, denoted bAP stim on the diagram)."

Is the stimulus used for Fig. 6 different? Give details.

Yes. Stimulus for Fig. 6 is a point input into the cytosol at the end of the dendrite. This information has been added to updated Fig. 2 and mentioned in the caption of Fig 2:

"*For simulating synaptic activation, calcium molecules were injected into submembrane voxels in the middle of the dendritic morphology (denoted synaptic stim on the diagram). For*

simulating b-AP like stimulus, calcium molecules were injected into the cytosol to an end dendritic voxel (point stimulus, denoted bAP stim on the diagram)."

and in the Methods section, lines 151-157:

"*We simulated two types of calcium influx to the dendrite (Fig 2)*

1. synaptic-like activation as a 40 ms influx of calcium molecules into submembrane voxels in the middle of the dendrite (summary of the stimulation is presented in Tab. 9),

2. bAP-like stimulus as a 3 ms influx of calcium molecules into the cytosol voxels at the end of the dendrite similarly as in [23] (summary of the stimulation is presented in Tab. 10)."

Line 116: Much more detail about simulations and their analysis is needed. How many simulations are run to collect the values shown in the figures? Where are measurements made? What do the error bars show, and why are there not horizontal error bars showing variations in peak amplitude? Statistical analysis showing significance of differences between the different modelled situations is required to support the qualitative description of the results given. Why is spatial extent measured as distance at which amplitude drops below 250% of resting level?

We rewrote the Methods section and added a detailed description of stimulation (methods section titled "Stimulation protocols") and analysis (titled "Analysis", lines 182-218).

The error bars show standard error calculated from 10 trials (random seeds), except for Fig 8 where we explicitly state that we plot standard deviation. The horizontal error bars do not appear because they are smaller than the data points on the plots. A statement to this effect has been added to Figures 3,and 4.

We measured spatial extent as distance at which calcium amplitude exceeds 250 % of mean intracellular concentration. Calcium waves are defined as contiguous clusters of calcium events in space and time, in which the calcium exceeds the threshold of calcium concentration. We chose 250% of resting calcium concentration to detect low calcium elevations and to avoid noise fluctuations due to the stochastic simulations.

We have now used ANCOVA to analyze the differences in the models, one analysis for each panel in figures 3, 5 and 6. These statistics are reported in Table 11 and in the text. The analysis is explained on lines 208-215.

Results

Some suggestions about improving the figures are given below.
As noted above, since all of these results come from stochastic simulations, the statistical significance of differences between simulated situations can and should be calculated.
Line 144: define explicitly what you mean by "spatial and temporal specificity" – presumably the spread of amplitude and decay time constant values?

Following reviewer 2's suggestion we removed from the manuscript results of the model with RyR2 localized in the dendritic membrane. We added a definition of spatial specificity and temporal specificity introduction (lines 56-58): "*Furthermore, we show that disinhibition of RyR2 increases spatial spread of calcium transients (lowers spatial specificity) and increases temporal decay of calcium transients (decreases temporal specificity) evoked by CICR in the dendrite.*" and explained how we calculated spatial spread and decay time in the Methods / Analysis (lines 183-192).

Line 167: more discussion is needed of what Fig. 6 is showing that is different from Figs. 4 and 5; what is different about these simulations and why do you only refer to these results re lack of calcium wave propagation (since figs. 4 and 5 indicated this too)?

Fig. 6 (Fig 4 in the revised manuscript) demonstrates that the inhibition of RyR2 by calmodulin does not block calcium wave propagation. It also uses the bAP-like stimulus, to show that the observed results (lack of calcium waves and increased spatial extent with disinhibition of RyR2) are independent of the type of calcium input. We now explain this better in the results (lines 243-247) and updated the Figure caption.

Line 168: what is Figure 7 for? Surely the same conclusions are already drawn before and this is just a different way of plotting the same data?

We have removed figure 7 from the manuscript.

Line 200: indicate to the reader that only results for the thin dendrite are shown in fig. 9

We now state that we were showing results only for thin dendrites in the caption of Fig 6 in the revised manuscript version.

Line 248: change text to "for the situations summarized in Fig. 10";

We changed this text to: "*Cases summarized in Fig 7*" (line 256)

how do the calcium dynamics illustrated in Fig. 11 (sparks and waves) impact on the data presented in Fig. 10: Surely the time varying amplitudes must affect the average calcium concentrations presented in fig. 10 (how were these measured? At a point in time and space, or across all time points?)? Do these dynamics cause the large increases in variance seen in Fig. 10 (ie is this what you mean in line 244?)?

Yes, the calcium sparks and waves do impact the data presented in Fig. 10 (Fig 8 in the revised manuscript), because the mean and variance of the intracellular calcium concentration (Fig. 10, Fig. 8 in the revised manuscript) were calculated across the whole dendritic morphology for the whole simulation. However, the calcium sparks and waves affected mostly the variance and not the mean. The evidence for this is that the 80% PMCA,

100% RyR2Cam, 0% RyR2 case had no calcium events (as seen in Fig 9a) but did have an elevated mean calcium (Fig 8).

I am confused as to the difference between the results of Fig. 12 and those of Fig. 13a: this needs clarifying in the text.

We do agree that this is confusing. We had originally defined a calcium wave as a calcium event that propagated throughout the dendrite, but realized that this “50 um threshold” was somewhat arbitrary. We now state in the manuscript that some of the calcium events are calcium waves (line 361), and have eliminated Figure 13. Fig 9a shows frequency of all events, and from the spatial extent shown in Fig 9d it is apparent that the events for some of the conditions are actually calcium waves.

Line 260-262: what exactly do you mean by “widest” and “longest” calcium waves? Ie what are the widths and lengths that you are measuring?

We changed the width to duration and length to spread in the figures and manuscript. We defined calcium events as elevations of calcium concentration higher than 250% of the mean resting intracellular concentration of the control model (76 nM). The widest calcium waves were those with the largest duration of the calcium elevation exceeding 250 % of 76 nM, whereas the longest were the ones with the largest spatial spread of calcium events (measured as the length of the part of the dendritic morphology, where the calcium elevation exceeded 250% of the mean resting intracellular concentration of the control mode). We no longer talk about widest and longest and instead specify longest duration and largest spatial spread (lines 367-368).

Line 283: and now, what do you mean by “CICR distance”? Explain this, or change back to previously used terminology eg spatial extent.

We changed “CICR distance” to “*change in spatial extent of calcium elevations*”, now in line 383.

Line 287: why is this unexpected?

You were right it is expected that PMCA would play the largest role in the thin and medium dendrites. We removed the word “Unexpectedly”.

Discussion

After the opening sentence, discuss more the evidence for the role(s) dendritic calcium might play in learning and memory, citing suitable references (such as [4] and [87]; any others?) Include brief discussion of RyRs in spines (RyR3 activated by calmodulin, rather than inhibited) and the role CICR might play in spine heads, as opposed to in dendritic shafts. Then start a new paragraph with “In this work…”

We rewrote the Discussion to summarize our results in the 1st paragraph, and then discuss the role of RyR and dendritic calcium in learning and memory in the 2nd paragraph, which also discusses RyRs in spines. The subsequent paragraph then compares our results with

published work. The relevant paragraph from the discussion now reads as follows (lines 404-418):

“The influence of calmodulin and RyR2 on calcium dynamics is critical because learning, memory and many other processes in the brain are controlled by calcium dynamics. Dendritic calcium plays a crucial role in behavioral time scale plasticity, a very powerful form of synaptic plasticity requiring a prolonged dendritic calcium elevation, which underlies tuning of spatial features in dendrites and is capable of producing place cells [99]. Interestingly, blocking mitochondrial calcium buffering, and hence increasing calcium release from the ER, led to higher stability of place cells [100]. Dendritic calcium is strongly correlated with spine calcium [83] with RyR3s (activated by calmodulin) on the spine endoplasmic reticulum (spine apparatus) transducing spine calcium signals to the dendrite [101, 102]. Calmodulin acting on RyRs differentially regulates the calcium transients in the dendritic branch: leading to an amplification of calcium transients from individual spines and restricting their spread in the dendrite leading to larger spatial and temporal specificity of calcium signaling in the dendrite. Thus, RyRs can influence learning.”

Line 317 onwards: start new paragraph at “Although”; add “methodological differences from [23]”; add more text here describing where low copy numbers occur in your simulations that mean the use of a stochastic model is important and indicate what results may depend on this ie expand on your comment that predictions may depend on this.

We now have a separate paragraph on some of the methodological differences between our work and other publications. We also added text explaining why stochastic simulations are important. This paragraph now reads (lines 430-451):

“Although, we used similar RyR2 densities (4 μm−2) in our model, there were many methodological differences from [23]: instead of using a deterministic model we used a stochastic approach for simulation of reaction-diffusion systems, which is better suited to dynamical systems with low copy numbers [103] and in such systems deterministic models might yield different predictions than stochastic models [104]. One of the differences is that deterministic rate laws depend on concentrations whereas stochastic rate laws depend on the number of possible combinations of reacting species. With high copy numbers stochastic and deterministic models will yield similar results, whereas with low copy numbers the reactions might differ substantially, e.g. contributing to higher noise sensitivity of deterministic models [104]. The stochastic approach we used is a spatial extension of Gillespie’s tau leap algorithm, which dynamically selects between simulating an exact event or a leap event both for reactions and diffusion between voxels [84]. This algorithm provides the computational efficiency needed for simulating this mathematically stiff system. Finally, we used a stochastic model of RyR2 activation [37] tuned to match mean open and close time of the RyR2 [39] instead of using the Keizer-Levine model of RyR2 activation [105], which exhibits very long RyR2 open times. It is possible that the induction of intracellular calcium waves in [23] resulted from using the Keizer-Levine RyR2 model. In fact, when we performed stochastic simulations with the model of resting dynamics, we observed spontaneous, oscillatory generation of intracellular calcium waves in thin dendrite. We did

not compare predictions of our model with [28] and [29], because both models implemented IP3 -dependent calcium wave generation."

Line 379: clarify if you are referring to experimental data or to your simulations when you refer to emergence of spontaneous calcium events in this sentence.

This line has been rewritten (along with surrounding sentences), and we start that sentence with "In our model". This section now reads (lines 494-498): **"***Increasing oxidation levels leads to both lower PMCA activity [109] and RyR2 disinhibition [6, 16]. In our model, lower PMCA activity alone resulted in the elevation of intracellular calcium concentration. Coupling the lower PMCA activity with disinhibited RyR2 caused the emergence of spontaneous calcium events, with frequency dependent on dendritic diameter and disinhibited RyR2 concentration."*

Figures

In general, figure captions should specify what is being shown in the figure, rather than give a description of the results, which is covered in the main text (see my comment on fig. 4, but it applies to most of the figure captions).
Figure 1: caption needs more detail about what is shown in the figure; the figure itself needs to be clearer as to the relationship between ER and the dendrite, and which is the dendritic (cellular) membrane and which the ER membrane.

We have added a detailed description of activation of signaling pathways depicted in Fig. 1, which now reads:

"*Molecular pathways implemented in the model. Calcium flows into the cell through calcium permeable channels in the cell membrane (denoted VGCC in the diagram and Leak in Tab. 2). Calcium is extruded into the extracellular space by plasma membrane Ca2+ -ATPase (PMCA) and sodium calcium exchanger (NCX) and into the endoplasmic reticulum by Sarcoendoplasmic Reticulum Calcium ATPase (SERCA). In the cytosol calcium is buffered by calbindin, indiffusible buffer (Buffer) and calmodulin (CaM). Apo-calmodulin (unbound to calcium) is buffered by neurogranin (Ng). Calcium flows from the ER to the cytosol through open ryanodine type 2 receptors (RyR2), which are activated by calcium and inhibited by calmodulin. In the ER calcium is buffered by calreticulin (CRT) and binds stromal interaction molecules (STIM), which serve as luminal calcium sensors. When ER in the endoplasmic reticulum is too low, STIM molecules form dimers and bind calcium release-activated calcium channel protein 1 (Orai1) opening them and allowing calcium from the extracellular space to flow into the cytosol, where it would be extruded by SERCA to the endoplasmic reticulum replenishing it. The ORA1-STIM channels constitute store-operated calcium entry (SOCE).*"

Figure 2: caption and/or figure could have more detail on subcompartment divisions (sizes).

We have added more details on the model's morphology. Caption of Fig. 2 now reads:

"*Fig 2. "Dendritic morphologies used in simulations. We used three dendritic morphologies: thin dendrite (diameter of 1.2 μm), medium dendrite (diameter of 2.4 μm) and thick dendrite (diameter of 6.0 μm). All dendrites were 51 μm long. Each morphology was subdivided into voxels, allowing two-dimensional diffusion. Submembrane voxels were cubes of 0.5 μm × 0.4 μm × 0.6 μm (the same for all dendritic diameters). The submembrane voxels allow for placement of membrane associated molecules. Cytosolic voxels were the same as submembrane voxels for the 1.2 μm model, cubes of 0.5 μm × 0.46 μm × 0.6 μm for the 2.4 μm model and cubes of 0.5 μm × 0.4615 μm × 0.6 μm for the 6.0 μm model. The dotted lines show some of the lengthwise and widthwise subdivisions into voxels. For simulating synaptic activation, calcium molecules were injected into submembrane voxels in the middle of the dendritic morphology (denoted synaptic stim on the diagram). For simulating b-AP like stimulus, calcium molecules were injected into the cytosol to an end dendritic voxel (point stimulus, denoted bAP stim on the diagram)."*

Figure 3: could possibly be merged with Fig. 2 by including one example on the medium dendrite and the other on the thick dendrite.

We have removed this figure following reviewer 2's suggestions.

Figure 4: here, and other figures, caption should describe what is plotted (and clarifying how calcium stimulus amplitudes relate to the data points shown), and not repeat the analysis of the results which is already given in the main text.

For all figure captions, we added a sentence in the beginning of the caption to indicate the main results, followed by a description of what is plotted, e.g. ("*Spatial extent (a) and temporal decay (b) of calcium transients for model with RyR2s inhibited by CaM (RyR2CaM), disinhibited RyR2s (RyR2 no CaM) and model without RyR2s (no RyR2)*"). We removed much of the description of the main results in order to shorten the caption, but we left in brief descriptions of the main results because we wanted to make the manuscript accessible to readers that only read figures and captions and skip the results text.

Figure 5: this should be merged with Figure 4, as Fig. 4 only contains one situation that is not plotted on Fig. 5.

We merged Fig 4 with Fig 5 (Figure 3 in the revised manuscript)

Figure 6: caption needs further details of stimulation protocol, to distinguish results from Figs. 4 and 5

We have added more information to this caption, currently Fig 4. The caption now reads:

"*Fig 4. Spatial spread of calcium transients for a bAP-like stimulus. Only results for the thin dendrite are shown. A short calcium input (3 ms) was applied directly to the cytosol at one side of the dendrite as in [23] (also shown in Fig 2). Disinhibition of RyR2 by calmodulin (empty circles) increases spatial extent, but does not allow for calcium wave propagation.*

Spatial extent was the furthest location from the stimulated site where calcium amplitude following stimulation exceeded 250% of resting calcium concentration. The horizontal error bars do not appear because they are smaller than the data points on the plots."

Figure 7: surely this figure could simply be removed; if there is a genuine need to retain it then more information must be given in the caption about what is plotted here.

We have removed Fig 7.

Figure 8: these results could also possibly be merged with those of figs. 4 and 5 to create a single figure.

Fig 8 (Fig 5 in the revised manuscript) shows the change due to old age, whereas Figs 4 & 5 (Fig 3 & 4 in the revised manuscript) have been changed/combined to show the effect of RyR2 in the control model. Thus, combining these two would be messy and confusing. Instead, we combined Fig 8 & 9 into one figure (Fig 5) that both compares ctrl to old age, and shows the contribution of RyR2 to the old age effect.

Figure 9: caption should indicate only thin dendrites shown.

This is no longer applicable because the information for Fig 9 has been added to Fig 8 to create new Fig 5.

Minor corrections

There are a number of typographical errors. The Discussion, in particular, needs careful proofreading. Here are some errors that were noted:
Line 69: brackets around Table 4
Line 74: refer to fig. 3 here
Line 94: define SOCE here (if not done earlier) and check that all other acronyms are defined at first usage
Line 132: "can not" to "cannot"
Line 161: refer to Fig. 5 here (near "observed")
Line 223: here and throughout, "dyshomeostasis" to "dishomeostasis"
Line 232: here and throughout, "dysfunction" to "disfunction"
Line 255: (Fig. 12)
Line 264: some wording has gone missing here
Line 293: delete "the" after "investigated"
Line 300: "becomes" to "become" and "disinhibit" to "disinhibits"
Line 318: "instead using" to "instead of using"
Line 326: "with model" to "with the model"
Line 331: um to uM
Line 340: full stop at end of sentence
Line 372: "lead" to "leads"
Line 395: add "of calcium transient." after "specificity"

We implemented all proposed minor corrections.
