# Supplementary material for: Calmodulin controls spatial and temporal specificity of calcium-induced calcium release
Source: PLoS Comput Biol. 2026 Sep 10;22(9):e1013752. doi: 10.1371/journal.pcbi.1013752 (PMC13581220; doi:10.1371/journal.pcbi.1013752)
Supplement: Supplementary file 2 [file pcbi.1013752.s006.pdf]

Revision letter

“Calmodulin controls spatial and temporal specificity of calcium-induced calcium release”

Dear Editors and Reviewers,

We would like to thank you for your detailed reviews of our manuscript, which greatly helped to improve it. We have rewritten the manuscript according to your comments. Please find our response below.

Thank you for your consideration of this manuscript.

Sincerely,

Joanna Jędrzejewska-Szmek and Kim T Blackwell

Reviewer #1: **The manuscript has improved significantly. However, the statement that these changes “underlie changes in calcium dynamics that cause deficits in learning and memory” appears stronger than what the model directly supports. Since the model does not explicitly simulate learning or memory mechanisms, the authors should either soften the wording to indicate that the results may be associated with such deficits, or provide an additional analysis linking the modeled calcium signals to a downstream model of synaptic plasticity or another mechanism more directly tied to learning and memory, such as dynamic changes in the number of AMPA receptors in the postsynaptic membrane. Although there is experimental evidence that RyRs can influence learning and memory through their contribution to dendritic and spine calcium transients, this influence on learning and memory was not explicitly incorporated into the current model and should be made clear in the text.**

The last sentence of the abstract now reads: “Overall, our research demonstrates that changes in calmodulin that are associated with AD and aging, by regulation of RyR2 (in old age) and PMCA (in AD), underlie changes in calcium dynamics that **might** have consequences for learning and memory.”

Reviewer #2: Major Comment:

1) The paper has been greatly improved. However, one major concern needs to still be addressed. In Tables 2 and 8, comparing the Control to Old Age condition, two offsetting parameter changes are made to control plasma membrane calcium fluxes. The Leak into the cell is reduced for old age (corresponding to decreased L-VGCC channels), and the PMCA kf is reduced. These changes offset such that resting cytoplasmic calcium stays about the same. That’s OK. The concern comes when going from the Old Age to the Alzheimer’s (AD) conditions. Then, the PMCA kf stays reduced, but careful inspection of the table shows that the authors put the Leak parameter back to the control value for AD, rather than keeping it at the reduced Old Age value. This increase in Leak is actually why resting calcium increases for AD compared to old age, and why spontaneous calcium transients occur, in the AD model! (this is evident from thermodynamic grounds, increased resting calcium has to be mediated by plasma membrane parameter changes) The resting calcium increase in AD is not, as text elsewhere implies, because of any specific decrease of PMCA activity in the AD condition relative to the Old Age condition.

The lack of discussion of this increase in Leak is misleading to the reader, because most readers would assume that a simulation of AD, a disease that typically occurs in old age, would also include the L-VGCC reduction that is typical of old age. It’s hard to see this issue when it is just buried in the Methods. I didn’t find this important issue the first time I read the paper. It needs to be adequately described in the text of Results and/or Discussion. The specific Discussion statement on line 495, that "lower PMCA activity alone" increased resting calcium, is misleading and needs to be removed. One way to handle this issue could be, to suggest that the model is most appropriate for simulating early-onset AD, which may occur before the age-related decrease in L-VGCCs. In contrast, simulation of calcium dynamics specific to late-onset AD may in addition require, as you actually already describe to some extent towards the end of the Discussion, inclusion of beta-amyloid effects on plasma membrane calcium flux. Also check the text wording elsewhere in the paper to make

sure it does not imply that the model implements a PMCA decrease that is specific to AD (e.g. last two lines of Abstract, Discussion as noted above).

The change in calcium channel expression with old age is not entirely clear. Experimental work performed in Paul Landfield's laboratory indicated that L-type VGCCs in CA1 pyramidal cells of aged male rats are upregulated (Thibault and Landfield, 1996; Campbell et al., 1996; Thibault et al., 2001). Simultaneously, experimental evidence suggests that CACNA1g is downregulated in the human hippocampus and T-type VGCC expression is lower in female aged mice (Rice et al., 2014).

We agree that Leak balances PMCA and NCX activity to keep stable resting intracellular calcium. Also, its physiological interpretation is tied to the opening of VGCCs.. In the old age model, we lowered Leak because experimental evidence suggests that the resting intracellular calcium does not change as rats age (Gant et al., 2006). In the AD model, the reason for keeping the control value of Leak is to pinpoint which factors might be responsible for elevated resting intracellular calcium concentration, i.e., whether disinhibited RyR2 or lower PMCA kcat might be responsible for elevated resting intracellular calcium. However, we do agree that the AD model represents either the early, presymptomatic stages of AD or the early onset of AD, and we added that information to the appropriate sections (lines 140 and 322) and modified the Discussion statement in line 499:

"In our model of early onset AD, with unchanged resting calcium influx to the dendrite, lower PMCA activity alone resulted in the elevation of intracellular calcium concentration."

The statement in line 519 now reads:

"*It is important to note that the old age model and the AD model represent two different conditions. The old age model differs from the AD model in (a) the lack of SOCE, (b) higher buffering molecules, including neurogranin, and (c) unchanged calcium leak from the extracellular space, which represents resting calcium influx to the dendrite.*"

We also explicitly state that the early onset AD model has unchanged Leak in lines 145 and 328.

Thibault O, Landfield P. Increase in Single L-Type Calcium Channels in Hippocampal Neurons During Aging. Science. 1996;272(5264):1017-1020.

Campbell LW, Hao SY, Thibault O, Blalock EM, Landfield PW. Aging changes in voltage-gated calcium currents in hippocampal CA1 neurons. Journal of Neuroscience. 1996;16(19):6286–6295.

Thibault O, Hadley R, Landfield PW. Elevated postsynaptic [Ca2+]i and L-type calcium channel activity in aged hippocampal neurons: relationship to impaired synaptic plasticity. Journal of Neuroscience. 2001;21(24):9744–9756.

Gant JC, Sama MM, Landfield PW, Thibault O. Early and Simultaneous Emergence of Multiple Hippocampal Biomarkers of Aging Is Mediated by Ca2+-Induced Ca2+ Release. Journal of Neuroscience. 2006;26(13):3482-3490

Rice RA, Berchtold NC, Cotman CW, Green KN. Age-related downregulation of the CaV3.1 T-type calcium channel as a mediator of amyloid beta production. Neurobiology of Aging. 2014;35(5):1002–1011.

Minor Comments:
1) Abstract line 7, “relieving of” should be “relief of”
2) Line 57 (Introduction), and line 280 (Results). An “increased” or “higher” temporal decay means, to most readers, an increased rate of decay and therefore a decreased duration of calcium transients. This is the opposite of what you want to say here. In these conditions the temporal specificity is reduced and the durations of transients are increased. Thus the decay rate, or just the decay, is decreased or lowered.

We now state explicitly „increases duration of calcium transients”

Reviewer #3: **I thank the authors for the work they have done to satisfy all my comments and suggestions on the first draft.**
