# Supplementary material for: Calmodulin controls spatial and temporal specificity of calcium-induced calcium release
Source: PLoS Comput Biol. 2026 Sep 10;22(9):e1013752. doi: 10.1371/journal.pcbi.1013752 (PMC13581220; doi:10.1371/journal.pcbi.1013752)
Supplement: Peer review history

# Peer review: Decision Letter 0

**DOI:** 10.1371/journal.pcbi.1013752.r001

**Authors:** Ausra Saudargiene, Andrea E. Martin

26 Mar 2026

Calmodulin controls spatial and temporal specificity of calcium-induced calcium release

PLOS Computational Biology

Dear Dr. Joanna Jędrzejewska-Szmek,

Thank you for submitting your manuscript to PLOS Computational Biology. After careful consideration, we feel that it has merit but does not fully meet PLOS Computational Biology's publication criteria as it currently stands. Therefore, we invite you to submit a revised version of the manuscript that addresses the points raised during the review process.

Please submit your revised manuscript by 25 April. If you will need more time than this to complete your revisions, please reply to this message or contact the journal office at ploscompbiol@plos.org. When you're ready to submit your revision, log on to https://www.editorialmanager.com/pcompbiol/ and select the 'Submissions Needing Revision' folder to locate your manuscript file.

Please include the following items when submitting your revised manuscript:

* A letter that responds to each point raised by the editor and reviewer(s). You should upload this letter as a separate file labeled 'Response to Reviewers'. This file does not need to include responses to formatting updates and technical items listed in the 'Journal Requirements' section below.

* A marked-up copy of your manuscript that highlights changes made to the original version. You should upload this as a separate file labeled 'Revised Manuscript with Track Changes'.

* An unmarked version of your revised paper without tracked changes. You should upload this as a separate file labeled 'Manuscript'.

If you would like to make changes to your financial disclosure, competing interests statement, or data availability statement, please make these updates within the submission form at the time of resubmission. Guidelines for resubmitting your figure files are available below the reviewer comments at the end of this letter

We look forward to receiving your revised manuscript.

Kind regards,

Ausra Saudargiene, Ph.D.

Academic Editor

PLOS Computational Biology

Andrea E. Martin

Section Editor

PLOS Computational Biology

**Journal Requirements:**

1) Please ensure that the CRediT author contributions listed for every co-author are completed accurately and in full.

At this stage, the following Authors/Authors require contributions: Joanna Jędrzejewska-Szmek, and Kim T Blackwell. Please ensure that the full contributions of each author are acknowledged in the "Add/Edit/Remove Authors" section of our submission form.

The list of CRediT author contributions may be found here: https://journals.plos.org/ploscompbiol/s/authorship#loc-author-contributions

2) We ask that a manuscript source file is provided at Revision. Please upload your manuscript file as a .doc, .docx, .rtf or .tex. If you are providing a .tex file, please upload it under the item type u2018LaTeX Source Fileu2019 and leave your .pdf version as the item type u2018Manuscriptu2019.

**Reviewers' comments:**

Reviewer's Responses to Questions

**Comments to the Authors:**

**Please note here if the review is uploaded as an attachment.**

Reviewer #1: The investigation of CICR in control and experimental conditions -aging, AD- is relevant and original. The model explores different scenarios and investigates the role ER Ca2+ release, RyR2, PMCA and CaM in this process. However, the authors need to address some issues.

Justify why a stochastic model is really needed. Compare with a deterministic solution.

The work mention the role of Ca2+ in learning and memory, but the model does not reproduce learning an memory phenomena. How to truly link the model results with learning and memory?

Check throughout for typos and English errors. To mention some: ‘disinhibted’, pg. 9. ‘AD we tested …’, pg 12.

Check labels and titles in all figure: Peak Ca or peak Ca^2+? Ca or Ca^2+?

Make sure fonts are consistent in the figures.

Fig. 15: Paradigm is upside-down.

Fig 2: Use the proper superscript for 2+ in Ca2+

There is an overlap in the header of the tables 2, 7 and 8 between kf and kr.

Justify the inconsistent units between tables for kf. Table 4: kf(nM/ms) Table 2 kf(nM^-1 ms^-1). Table 6 kf(nM^4/ms). How are the different units handled in the program code?

Reviewer #2: Major Comments:

1) The text of the paper, including but not limited to the Discussion, needs to be revised to eliminate any implication that the authors are simulating any changes or processes specific to Alzheimer’s disease. Because, in fact they are not, all the listed changes in model parameters – altered RyR2-Cam binding, decreased PMCA pump activity, downregulation of SOCE and of T-type Ca channels – are generic to old age, not specific to AD. Indeed, some of the tables of model parameter values have columns listing old age values, but not columns listing different AD values. If the authors really want to claim they are simulating an AD condition, they need to extend the model and present additional simulations, by adding the effects of elevated beta-amyloid that they note in the Discussion.

2) The authors say their model is multi-compartment, but do not give or sufficiently justify a description of these compartments. How is their submembrane compartment different from the rest of the dendrite, why does it have the given width? Are there simple exchange terms from this compartment to the rest? Figure 3 has a very odd depiction of the dendrite as a gold cylinder hovering between two planar plasma membranes. I think the dendrite is instead supposed to be a box as in Fig. 2, so fix Fig. 3. Also, from the author’s preceding paper ref. 32, I guess the dendrite is divided into voxels. What is their size and shape? If NeuroRD does it automatically, still give an approximate description. Is diffusion modeled using the diffusion partial differential equation everywhere, or is it modeled by simple exchange terms at the boundaries of voxels with voxel interiors assumed homogenous, or is there both diffusion and exchange terms?

3) The paper implies, at first reading, that the ER is modeled as distinct internal compartments with their own surfaces and internal volume. Figure 3, right panel, illustrates the model as if this was the case. But for this model variant (I comment below on the left panel) It looks like the authors distributed the variables RyR2, RyR2Ca, and CaER uniformly throughout (according to lines 74-76) a quarter of the dendrite, with this quarter consisting of one or more compartments with RyR2 and RyR2Ca volume-distributed. The authors did not, it appears, construct separate ER compartments with their own surface putting RyR2 on the surface of these compartments. And, given that the authors do not mention separate CaER variables for separate compartments, I think they only assume one internal ER compartment, which is contrary to what the figure shows. It is essential that the article text, and the figure in particular the location of RyR2, SERCA.., be clarified, and extended as necessary, so that no misleading material is present in this regard. It would also be good to give some detail about how existing data-based ER reconstructions compare with this model.

4) The ER model in the left panel of Fig. 3, in which the RyR2s are all located “at the dendritic membrane”, by which is meant either the lower or upper face of the model’s submembrane compartment, seems unjustified. It looks like here the authors put a planar box, right below the submembrane compartment, taking up a quarter of the dendrite volume, with the RyRs and SERCA all on that upper face. In reality, even if an ER cisternae lies against the membrane, I don’t think the RyRs and SERCAs would all be on that face, they should be on other faces of the ER compartment as well. Is there relevant data, has anyone showed by immunostaining or another method how RyR2 or SERCA is distributed within the dendrite? Anyway, the authors state later that simulations with this model variant are not significantly different from simulations with the model variant in the right panel. Thus, I think that model variant should be removed.

5) The authors need to state if, and for how long, their model with basal parameters and starting from initial concentrations was equilibrated, prior to giving a stimulus.

6) References, or other justification for estimates, need to be provided for the parameter values in Tables 1, 3, and 5. For non-ER parameters some of this may already be in the authors’ preceding paper, Ref. 32. If so make it clear to the reader, describing for which parameters references or justification is found in Ref. 32. But for parameters new to this model it needs to be done here.

7) It is stated that the simulations are performed stochastically, with use of the Gillespie algorithm. Is this true even for the most abundant molecules and fastest events, like calcium binding and unbinding from buffers? What about calcium diffusion. It appears the authors likely use a hybrid simulation scheme where (some or all) reactions are Gillespie and diffusion (at least) is not?

8) Tables 6 and 7 repeatedly use the phrase “adjusted to effect”. Explain what this means. For example it could mean data shown in a particular figure of the referenced publication.

9) I see one place, Figure 7, where the authors give a calcium stimulus amplitude, but it is in molecules injected, which is hard to relate to physiological stimulus amplitudes as commonly reported. Please compare the amplitudes to physiological stimulus amplitudes in some manner. What is the duration? On line 110 the authors say the authors increased calcium stimulus to take into account L-type channel upregulation. How much? But in fact, in the Discussion it says L type VGCCs were “not included in the model”? What about the T-type channel downregulation the authors say also occurs in aging?

10) During each simulation in the old age condition, when RyRs are only 50% saturated with CaM, this level of saturation is not a dynamic variable. In actuality, though, this level should vary because the binding of calcium to CaM is dynamic, calcium is varying, and apo-CaM and Ca-bound CaM should have different RyR2 affinities. If the authors don’t want to add simulations, they need to acknowledge in the Discussion that this is a model limitation, or else justify why it can be neglected. I guess if half of the RyR2s are actually normal and half can’t bind CaM at all, then it could be neglected, but this should be better justified.

11) For Figure 1, the legend is not adequate. I’m not sure this figure shows all the pathways, given that this model builds on that presented in Ref. 32. In the previous Ref. 32 the authors have another similar figure of signaling pathways. Tell the reader; if they combine that figure with this Fig. 1, do they see all the pathways?

12) Lines 275-277, there is a sentence, “This result is consistent with experiments showing that in hippocampal CA1 neurons IP3-sensitive calcium store refilling required ORAI1 channels, whereas ryanodine-sensitive calcium store refilling involved VGCCs”. This sentence is not justified, because none of the simulations in this paper were designed to specifically examine depletion of calcium stores or mechanisms for their refilling. Either remove this sentence or add additional, appropriate simulations carried out for a longer time during which ORA1I and VGCCs are separately inhibited.

13) Lines 79-82 as written imply two different models were used to describe the RyR2. This needs to be better explained. How is the model for the oxidized state (the Keizer Smith model) different from the model for the non-oxidized state? Can this difference be justified?

Minor Comments:

1) In Fig. 11b, it looks like the calcium waves always start at one end of the dendrite. Why don’t some initiate in the middle, like the calcium sparks do?

2) Tables should be numbered in the order in which they are introduced in the manuscript, and should be briefly described when first introduced (this is not done for Table 1).

3) The legend of Fig. 2 is not adequate. Give dimensions, including the submembrane compartment. The legend of Fig. 3 needs to be revised because the model is not actually implementing “parallel cisternae” as compartments with distinct surfaces.

4) There are a number of minor grammar errors throughout the manuscript, go over it with either a human or AI editor.

5) Line 145, Fig. 4 should be Fig. 3.

6) The acronyms EPJ and SOCE need to be defined at first use.

7) Is “spatial extent” calculated for Fig. 5 the same as for Fig. 6?

8) The formatting of the References has numerous inconsistencies.

9) Lines 138-140, you say 4 um is a theoretical limit for calcium transient spread. I don’t see how this can be a theoretical limit, it must be parameter value dependent.

Reviewer #3: This manuscript describes the use of a computational model to explore the contribution of calcium-induced calcium release (CICR) from endoplasmic reticulum (ER) to calcium transients and resting state in neuronal dendrites. Calcium dynamics are crucial to neuronal function and synaptic plasticity, so understanding the many mechanisms that determine such dynamics is highly important. This current work provides a useful increment to our knowledge, detailing the amplitude, spatial extent and time course of dendritic calcium transients due to pseudo-synaptic stimulation, and the contribution of CICR to this. The effects of changes in CICR during aging and Alzheimer’s disease are explored. The presentation of the work, however, could do with some improvement, as detailed below.

Introduction

Point the reader to Fig. 1 when ER first introduced eg around line 14.

Methods

Line 111: Details of the calcium stimuli used are needed (amplitudes, time course). Are these stimuli reasonable approximations to what is expected from synaptic activation on a spine head? Does figure 2 indicate that the stimuli are from a point source at the location indicated? Is the stimulus used for Fig. 6 different? Give details.

Line 116: Much more detail about simulations and their analysis is needed. How many simulations are run to collect the values shown in the figures? Where are measurements made? What do the error bars show, and why are there not horizontal error bars showing variations in peak amplitude? Statistical analysis showing significance of differences between the different modelled situations is required to support the qualitative description of the results given. Why is spatial extent measured as distance at which amplitude drops below 250% of resting level?

Results

Some suggestions about improving the figures are given below.

As noted above, since all of these results come from stochastic simulations, the statistical significance of differences between simulated situations can and should be calculated.

Line 144: define explicitly what you mean by “spatial and temporal specificity” – presumably the spread of amplitude and decay time constant values?

Line 167: more discussion is needed of what Fig. 6 is showing that is different from Figs. 4 and 5; what is different about these simulations and why do you only refer to these results re lack of calcium wave propagation (since figs. 4 and 5 indicated this too)?

Line 168: what is Figure 7 for? Surely the same conclusions are already drawn before and this is just a different way of plotting the same data?

Line 200: indicate to the reader that only results for the thin dendrite are shown in fig. 9

Line 248: change text to “for the situations summarized in Fig. 10”; how do the calcium dynamics illustrated in Fig. 11 (sparks and waves) impact on the data presented in Fig. 10: Surely the time varying amplitudes must affect the average calcium concentrations presented in fig. 10 (how were these measured? At a point in time and space, or across all time points?)? Do these dynamics cause the large increases in variance seen in Fig. 10 (ie is this what you mean in line 244?)?

I am confused as to the difference between the results of Fig. 12 and those of Fig. 13a: this needs clarifying in the text.

Line 260-262: what exactly do you mean by “widest” and “longest” calcium waves? Ie what are the widths and lengths that you are measuring?

Line 283: and now, what do you mean by “CICR distance”? Explain this, or change back to previously used terminology eg spatial extent.

Line 287: why is this unexpected?

Discussion

After the opening sentence, discuss more the evidence for the role(s) dendritic calcium might play in learning and memory, citing suitable references (such as [4] and [87]; any others?) Include brief discussion of RyRs in spines (RyR3 activated by calmodulin, rather than inhibited) and the role CICR might play in spine heads, as opposed to in dendritic shafts. Then start a new paragraph with “In this work…”

Line 317 onwards: start new paragraph at “Although”; add “methodological differences from [23]”; add more text here describing where low copy numbers occur in your simulations that mean the use of a stochastic model is important and indicate what results may depend on this ie expand on your comment that predictions may depend on this.

Line 379: clarify if you are referring to experimental data or to your simulations when you refer to emergence of spontaneous calcium events in this sentence.

Figures

In general, figure captions should specify what is being shown in the figure, rather than give a description of the results, which is covered in the main text (see my comment on fig. 4, but it applies to most of the figure captions).

Figure 1: caption needs more detail about what is shown in the figure; the figure itself needs to be clearer as to the relationship between ER and the dendrite, and which is the dendritic (cellular) membrane and which the ER membrane.

Figure 2: caption and/or figure could have more detail on subcompartment divisions (sizes).

Figure 3: could possibly be merged with Fig. 2 by including one example on the medium dendrite and the other on the thick dendrite.

Figure 4: here, and other figures, caption should describe what is plotted (and clarifying how calcium stimulus amplitudes relate to the data points shown), and not repeat the analysis of the results which is already given in the main text.

Figure 5: this should be merged with Figure 4, as Fig. 4 only contains one situation that is not plotted on Fig. 5.

Figure 6: caption needs further details of stimulation protocol, to distinguish results from Figs. 4 and 5

Figure 7: surely this figure could simply be removed; if there is a genuine need to retain it then more information must be given in the caption about what is plotted here.

Figure 8: these results could also possibly be merged with those of figs. 4 and 5 to create a single figure.

Figure 9: caption should indicate only thin dendrites shown.

Minor corrections

There are a number of typographical errors. The Discussion, in particular, needs careful proofreading. Here are some errors that were noted:

Line 69: brackets around Table 4

Line 74: refer to fig. 3 here

Line 94: define SOCE here (if not done earlier) and check that all other acronyms are defined at first usage

Line 132: “can not” to “cannot”

Line 161: refer to Fig. 5 here (near “observed”)

Line 223: here and throughout, “dyshomeostasis” to “dishomeostasis”

Line 232: here and throughout, “dysfunction” to “disfunction”

Line 255: (Fig. 12)

Line 264: some wording has gone missing here

Line 293: delete “the” after “investigated”

Line 300: “becomes” to “become” and “disinhibit” to “disinhibits”

Line 318: “instead using” to “instead of using”

Line 326: “with model” to “with the model”

Line 331: um to uM

Line 340: full stop at end of sentence

Line 372: “lead” to “leads”

Line 395: add “of calcium transients” after “specificity”

**********

**Have the authors made all data and (if applicable) computational code underlying the findings in their manuscript fully available?**

The PLOS Data policy requires authors to make all data and code underlying the findings described in their manuscript fully available without restriction, with rare exception (please refer to the Data Availability Statement in the manuscript PDF file). The data and code should be provided as part of the manuscript or its supporting information, or deposited to a public repository. For example, in addition to summary statistics, the data points behind means, medians and variance measures should be available. If there are restrictions on publicly sharing data or code —e.g. participant privacy or use of data from a third party—those must be specified.

Reviewer #1: Yes

Reviewer #2: Yes

Reviewer #3: Yes

**********

PLOS authors have the option to publish the peer review history of their article (what does this mean?). If published, this will include your full peer review and any attached files.

If you choose “no”, your identity will remain anonymous but your review may still be made public.

**Do you want your identity to be public for this peer review?**  For information about this choice, including consent withdrawal, please see our Privacy Policy.

Reviewer #1: No

Reviewer #2: No

Reviewer #3: No

[NOTE: If reviewer comments were submitted as an attachment file, they will be attached to this email and accessible via the submission site. Please log into your account, locate the manuscript record, and check for the action link "View Attachments". If this link does not appear, there are no attachment files.]

**Figure resubmission:**

After uploading your figures to PLOS’s NAAS tool - https://ngplosjournals.pagemajik.ai/artanalysis, NAAS will process the files provided and display the results in the "Uploaded Files" section of the page as the processing is complete. If the uploaded figures meet our requirements (or NAAS is able to fix the files to meet our requirements), the figure will be marked as "fixed" above. If NAAS is unable to fix the files, a red "failed" label will appear above. When NAAS has confirmed that the figure files meet our requirements, please download the file via the download option, and include these NAAS processed figure files when submitting your revised manuscript.

**Reproducibility:**

?>

# Author comment: Author response to Decision Letter 1

**DOI:** 10.1371/journal.pcbi.1013752.r002

19 May 2026

Attachment Submitted filename: Revision letter (1).pdf

# Peer review: Decision Letter 1

**DOI:** 10.1371/journal.pcbi.1013752.r003

**Authors:** Ausra Saudargiene, Andrea E. Martin

7 Jul 2026

PCOMPBIOL-D-25-02396R1

Calmodulin controls spatial and temporal specificity of calcium-induced calcium release

PLOS Computational Biology

Dear Dr. Joanna Jędrzejewska-Szmek,

Thank you for submitting your manuscript to PLOS Computational Biology. After careful consideration, we feel that it has merit but does not fully meet PLOS Computational Biology's publication criteria as it currently stands. Therefore, we invite you to submit a revised version of the manuscript that addresses the points raised during the review process.

Please submit your revised manuscript by 7 August 2026. If you will need more time than this to complete your revisions, please reply to this message or contact the journal office at ploscompbiol@plos.org. When you're ready to submit your revision, log on to https://www.editorialmanager.com/pcompbiol/ and select the 'Submissions Needing Revision' folder to locate your manuscript file.

Please include the following items when submitting your revised manuscript:

* A letter that responds to each point raised by the editor and reviewer(s). You should upload this letter as a separate file labeled 'Response to Reviewers'. This file does not need to include responses to formatting updates and technical items listed in the 'Journal Requirements' section below.

* A marked-up copy of your manuscript that highlights changes made to the original version. You should upload this as a separate file labeled 'Revised Manuscript with Track Changes'.

* An unmarked version of your revised paper without tracked changes. You should upload this as a separate file labeled 'Manuscript'.

If you would like to make changes to your financial disclosure, competing interests statement, or data availability statement, please make these updates within the submission form at the time of resubmission. Guidelines for resubmitting your figure files are available below the reviewer comments at the end of this letter.

As the corresponding author, your ORCID iD is verified in the submission system and will appear in the published article. PLOS supports the use of ORCID, and we encourage all coauthors to register for an ORCID iD and use it as well. Please encourage your coauthors to verify their ORCID iD within the submission system before final acceptance, as unverified ORCID iDs will not appear in the published article. Only the individual author can complete the verification step; PLOS staff cannot verify ORCID iDs on behalf of authors.

We look forward to receiving your revised manuscript.

Kind regards,

Ausra Saudargiene, Ph.D.

Academic Editor

PLOS Computational Biology

Andrea E. Martin

Section Editor

PLOS Computational Biology

**Additional Editor Comments (if provided):**

**Journal Requirements:**

If the reviewer comments include a recommendation to cite specific previously published works, please review and evaluate these publications to determine whether they are relevant and should be cited. There is no requirement to cite these works unless the editor has indicated otherwise.

1)We have noticed that you have uploaded Supporting Information files, but you have not included a list of legends. Please add a full list of legends for your Supporting Information files after the references list

**Reviewers' comments:**

Reviewer's Responses to Questions

**Comments to the Authors:**

**Please note here if the review is uploaded as an attachment.**

Reviewer #1: The manuscript has improved significantly. However, the statement that these changes “underlie changes in calcium dynamics that cause deficits in learning and memory” appears stronger than what the model directly supports. Since the model does not explicitly simulate learning or memory mechanisms, the authors should either soften the wording to indicate that the results may be associated with such deficits, or provide an additional analysis linking the modeled calcium signals to a downstream model of synaptic plasticity or another mechanism more directly tied to learning and memory, such as dynamic changes in the number of AMPA receptors in the postsynaptic membrane. Although there is experimental evidence that RyRs can influence learning and memory through their contribution to dendritic and spine calcium transients, this influence on learning and memory was not explicitly incorporated into the current model and should be made clear in the text.

Reviewer #2: Major Comment:

1) The paper has been greatly improved. However, one major concern needs to still be addressed. In Tables 2 and 8, comparing the Control to Old Age condition, two offsetting parameter changes are made to control plasma membrane calcium fluxes. The Leak into the cell is reduced for old age (corresponding to decreased L-VGCC channels), and the PMCA kf is reduced. These changes offset such that resting cytoplasmic calcium stays about the same. That’s OK. The concern comes when going from the Old Age to the Alzheimer’s (AD) conditions. Then, the PMCA kf stays reduced, but careful inspection of the table shows that the authors put the Leak parameter back to the control value for AD, rather than keeping it at the reduced Old Age value. This increase in Leak is actually why resting calcium increases for AD compared to old age, and why spontaneous calcium transients occur, in the AD model! (this is evident from thermodynamic grounds, increased resting calcium has to be mediated by plasma membrane parameter changes) The resting calcium increase in AD is not, as text elsewhere implies, because of any specific decrease of PMCA activity in the AD condition relative to the Old Age condition.

The lack of discussion of this increase in Leak is misleading to the reader, because most readers would assume that a simulation of AD, a disease that typically occurs in old age, would also include the L-VGCC reduction that is typical of old age. It’s hard to see this issue when it is just buried in the Methods. I didn’t find this important issue the first time I read the paper. It needs to be adequately described in the text of Results and/or Discussion. The specific Discussion statement on line 495, that "lower PMCA activity alone" increased resting calcium, is misleading and needs to be removed.

One way to handle this issue could be, to suggest that the model is most appropriate for simulating early-onset AD, which may occur before the age-related decrease in L-VGCCs. In contrast, simulation of calcium dynamics specific to late-onset AD may in addition require, as you actually already describe to some extent towards the end of the Discussion, inclusion of beta-amyloid effects on plasma membrane calcium flux. Also check the text wording elsewhere in the paper to make sure it does not imply that the model implements a PMCA decrease that is specific to AD (e.g. last two lines of Abstract, Discussion as noted above).

Minor Comments:

1) Abstract line 7, “relieving of” should be “relief of”

2) Line 57 (Introduction), and line 280 (Results). An “increased” or “higher” temporal decay means, to most readers, an increased rate of decay and therefore a decreased duration of calcium transients. This is the opposite of what you want to say here. In these conditions the temporal specificity is reduced and the durations of transients are increased. Thus the decay rate, or just the decay, is decreased or lowered.

Reviewer #3: I thank the authors for the work they have done to satisfy all my comments and suggestions on the first draft.

**********

**Have the authors made all data and (if applicable) computational code underlying the findings in their manuscript fully available?**

The PLOS Data policy requires authors to make all data and code underlying the findings described in their manuscript fully available without restriction, with rare exception (please refer to the Data Availability Statement in the manuscript PDF file). The data and code should be provided as part of the manuscript or its supporting information, or deposited to a public repository. For example, in addition to summary statistics, the data points behind means, medians and variance measures should be available. If there are restrictions on publicly sharing data or code —e.g. participant privacy or use of data from a third party—those must be specified.

Reviewer #1: None

Reviewer #2: Yes

Reviewer #3: Yes

**********

PLOS authors have the option to publish the peer review history of their article (what does this mean?). If published, this will include your full peer review and any attached files.

If you choose “no”, your identity will remain anonymous but your review may still be made public.

**Do you want your identity to be public for this peer review?**  For information about this choice, including consent withdrawal, please see our Privacy Policy.

Reviewer #1: No

Reviewer #2: No

Reviewer #3: No

[NOTE: If reviewer comments were submitted as an attachment file, they will be attached to this email and accessible via the submission site. Please log into your account, locate the manuscript record, and check for the action link "View Attachments". If this link does not appear, there are no attachment files.]

**Figure resubmission:**

**Reproducibility:**

To enhance the reproducibility of your results, we recommend that authors of applicable studies deposit laboratory protocols in protocols.io, where a protocol can be assigned its own identifier (DOI) such that it can be cited independently in the future. Additionally, PLOS ONE offers an option to publish peer-reviewed clinical study protocols. Read more information on sharing protocols at https://plos.org/protocols?utm_medium=editorial-email&utm_source=authorletters&utm_campaign=protocols

# Author comment: Author response to Decision Letter 2

**DOI:** 10.1371/journal.pcbi.1013752.r004

22 Jul 2026

Attachment Submitted filename: Revision letter 2.pdf

# Peer review: Decision Letter 2

**DOI:** 10.1371/journal.pcbi.1013752.r005

**Authors:** Ausra Saudargiene, Andrea E. Martin

20 Aug 2026

Dear Dr Joanna Jędrzejewska-Szmek,

We are pleased to inform you that your manuscript 'Calmodulin controls spatial and temporal specificity of calcium-induced calcium release' has been provisionally accepted for publication in PLOS Computational Biology.

Before your manuscript can be formally accepted you will need to complete some formatting changes, which you will receive in a follow up email. A member of our team will be in touch with a set of requests.

Please note that your manuscript will not be scheduled for publication until you have made the required changes, so a swift response is appreciated.

IMPORTANT: The editorial review process is now complete. PLOS will only permit corrections to spelling, formatting or significant scientific errors from this point onwards. Requests for major changes, or any which affect the scientific understanding of your work, will cause delays to the publication date of your manuscript.

Should you, your institution's press office or the journal office choose to press release your paper, you will automatically be opted out of early publication. We ask that you notify us now if you or your institution is planning to press release the article. All press must be co-ordinated with PLOS.

Thank you again for supporting Open Access publishing; we are looking forward to publishing your work in PLOS Computational Biology.

Best regards,

Ausra Saudargiene, Ph.D.

Academic Editor

PLOS Computational Biology

Andrea E. Martin

Section Editor

PLOS Computational Biology

***********************************************************

Reviewer's Responses to Questions

**Comments to the Authors:**

**Please note here if the review is uploaded as an attachment.**

Reviewer #1: Thank you for the work that improved the manuscript. Minor: Please, replace the typo 'dishomeostasis' by 'dyshomeostasis' throughout the manuscript.

Reviewer #2: I am satisifed with these revisions, and believe the paper now makes a significant contribution to understanding mechanisms related to Alzheimer's disease pathology

**********

**Have the authors made all data and (if applicable) computational code underlying the findings in their manuscript fully available?**

The PLOS Data policy requires authors to make all data and code underlying the findings described in their manuscript fully available without restriction, with rare exception (please refer to the Data Availability Statement in the manuscript PDF file). The data and code should be provided as part of the manuscript or its supporting information, or deposited to a public repository. For example, in addition to summary statistics, the data points behind means, medians and variance measures should be available. If there are restrictions on publicly sharing data or code —e.g. participant privacy or use of data from a third party—those must be specified.

Reviewer #1: None

Reviewer #2: Yes

**********

PLOS authors have the option to publish the peer review history of their article (what does this mean?). If published, this will include your full peer review and any attached files.

If you choose “no”, your identity will remain anonymous but your review may still be made public.

**Do you want your identity to be public for this peer review?**  For information about this choice, including consent withdrawal, please see our Privacy Policy.

Reviewer #1: No

Reviewer #2: **Yes:** Paul Smolen

# Editor report: Acceptance letter

**DOI:** 10.1371/journal.pcbi.1013752.r006

**Authors:** Ausra Saudargiene, Andrea E. Martin

PCOMPBIOL-D-25-02396R2

Calmodulin controls spatial and temporal specificity of calcium-induced calcium release

Dear Dr Jędrzejewska-Szmek,

I am pleased to inform you that your manuscript has been formally accepted for publication in PLOS Computational Biology. Your manuscript is now with our production department and you will be notified of the publication date in due course.

The corresponding author will soon be receiving a typeset proof for review, to ensure errors have not been introduced during production. Please review the PDF proof of your manuscript carefully, as this is the last chance to correct any errors. Please note that major changes, or those which affect the scientific understanding of the work, will likely cause delays to the publication date of your manuscript.

Soon after your final files are uploaded, unless you have opted out, the early version of your manuscript will be published online. The date of the early version will be your article's publication date. The final article will be published to the same URL, and all versions of the paper will be accessible to readers.

For Research, Software, and Methods articles, you will receive an invoice from PLOS for your publication fee after your manuscript has reached the completed accept phase. If you receive an email requesting payment before acceptance or for any other service, this may be a phishing scheme. Learn how to identify phishing emails and protect your accounts at https://explore.plos.org/phishing.

Thank you again for supporting PLOS Computational Biology and open-access publishing. We are looking forward to publishing your work!

With kind regards,

Sharmila Kamatchi

PLOS Computational Biology | Carlyle House, Carlyle Road, Cambridge CB4 3DN | United Kingdom ploscompbiol@plos.org | Phone +44 (0) 1223-442824 | ploscompbiol.org | @PLOSCompBiol
